# P-44. Trend of Palliative Care Consult Among Patients with Staphylococcus aureus Bacteremia at a Mid-west Referral Center Between 2016 and 2018

**DOI:** 10.1093/ofid/ofaf695.273

**Published:** 2026-01-11

**Authors:** Mariana Kim Hsieh, Patrick M Mallea, Benjamin C Chen, Patrick Schwartzhoff, Alexandre Marra, Kunatum Prasidthrathsint, Yuya Hagiwara, Beth A Hanna, Jaime P Murphy, Paul G Auwaerter, Karen Brust, Takaaki Kobayashi

**Affiliations:** University of Iowa Health Care, Iowa City, IA; Oregon Health & Sciences University, Portland, Oregon; Loma Linda University, Loma Linda, California; University of Vermont Medical Center, Burlington, Vermont; University of Iowa Hospital and Clinics, iowa city, Iowa; University of Iowa Carver College of Medicine, Iowa City, Iowa; University of Iowa, Iowa City, Iowa; University of Iowa Health Care, Iowa City, IA; University of Iowa Health Care, Iowa City, IA; Johns Hopkins University School of Medicine, Lutherville, Maryland; University of Iowa Hospitals & Clinics, Iowa City, Iowa; University of Kentucky, Lexington, Kentucky

## Abstract

**Background:**

*Staphylococcus aureus* bacteremia (SAB) remains a leading cause of bloodstream infection in both community and healthcare settings, with reported mortality rates ranging from 10% to 30%. This study aimed to characterize the frequency, predictors, and clinical impact of palliative care consultation (PCC) in hospitalized patients with SAB.

**Figure 1.**
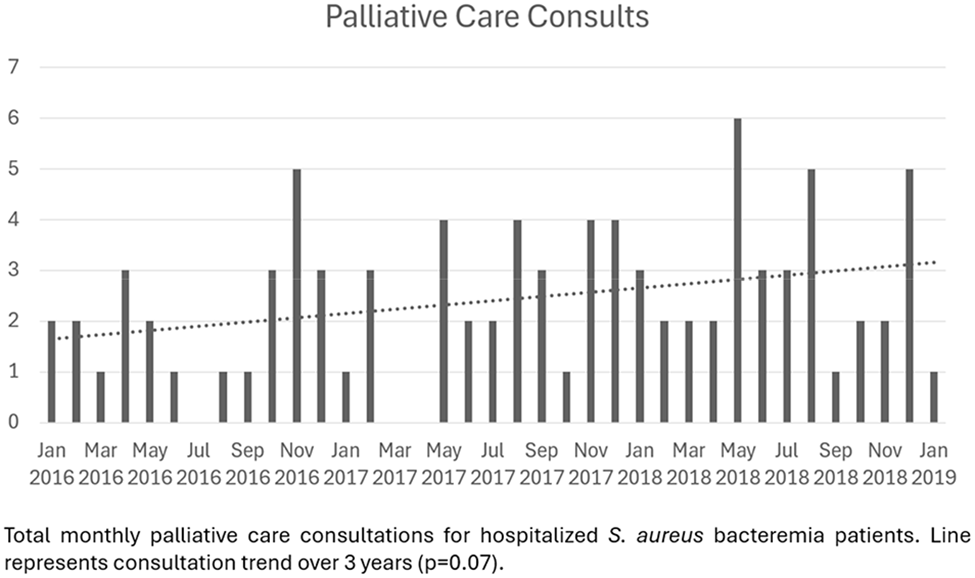
Distribution of Palliative Care Consults in hospitalized Staphylococcus aureus bacteremia patients

**Figure 2.**
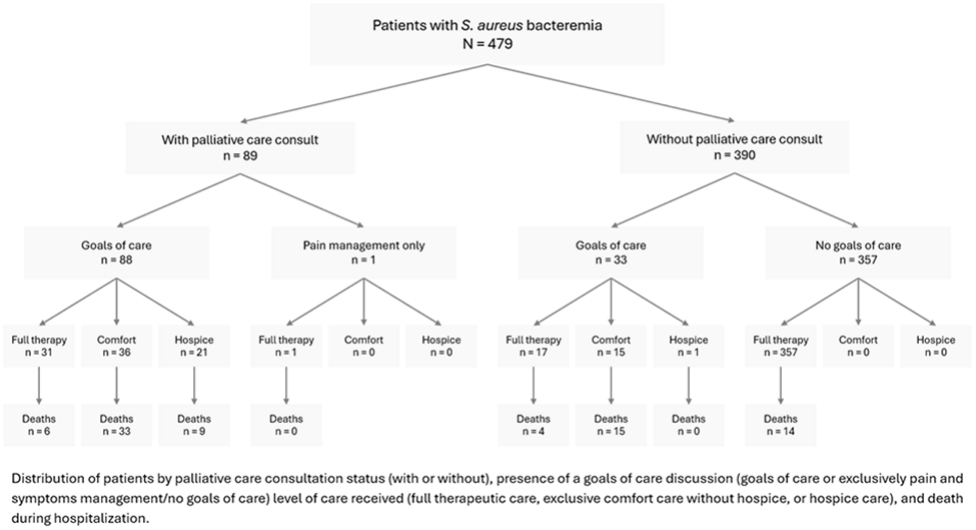
Distribution of Staphylococcus aureus bacteremia patients during hospitalization

**Methods:**

We conducted a retrospective cohort study of adult and pediatric inpatients with SAB, defined by ≥1 positive blood culture for *S. aureus*, between January 1, 2016, and December 31, 2018. Baseline characteristics were compared between patients who did and did not receive PCC using chi-square, Fisher’s exact, and Wilcoxon rank-sum tests. Temporal trends, predictors of PCC, and associated outcomes were assessed using multivariable regression models. Survival was analyzed using Kaplan-Meier methods.

**Results:**

Among 479 patients with SAB, 89 (18.6%) received PCC. Although PCC use increased over time, the trend was not statistically significant. Goals-of-care (GoC) discussions were the most common indication. Independent predictors of PCC included older age (adjusted odds ratio [aOR], 1.03 per year; 95% CI, 1.02–1.05; P< 0.001) and prolonged bacteremia (aOR, 1.10 per day; 95% CI, 1.00–1.21; P=0.042). Female sex (aOR, 0.54; 95% CI, 0.29–0.97; P=0.043) and musculoskeletal/soft tissue source of infection (aOR, 0.35; 95% CI, 0.14–0.83; P=0.021) were associated with lower odds of PCC. PCC was associated with shorter antibiotic duration (aOR, 0.58; 95% CI, 0.55–0.61; P< 0.001), increased GoC documentation (aOR, 1369.48; 95% CI, 257.29–25,917.74; P< 0.001), and higher rates of transition to comfort care (aOR, 42.40; 95% CI, 19.96–98.50; P< 0.001) and hospice (aOR, 164.34; 95% CI, 26.55–360.32; P< 0.001). Median time from consultation to discharge was 4 days (IQR, 1–10), and was shorter among those who died in-hospital (2 vs. 8 days; P< 0.0001).

**Conclusion:**

PCC was infrequently utilized among patients with SAB but strongly associated with care transitions and antimicrobial stewardship outcomes. A substantial proportion of patients died without PCC involvement. These findings highlight the need for earlier integration of palliative care in the management of SAB to support patient-centered care.

**Disclosures:**

Paul G. Auwaerter, MD, Capricor: Board Member|Capricor: Stocks/Bonds (Public Company)|Johnson and Johnson: Stocks/Bonds (Public Company)|Pfizer: Grant/Research Support|Shionogi: Advisor/Consultant

